# An Eco-Safety Assessment of Glyoxal-Containing Cellulose Ether on Freeze-Dried Microbial Strain, Cyanobacteria, *Daphnia*, and Zebrafish

**DOI:** 10.3390/ijerph14030323

**Published:** 2017-03-21

**Authors:** Chang-Beom Park, Min Ju Song, Nak Woon Choi, Sunghoon Kim, Hyun Pyo Jeon, Sanghun Kim, Youngjun Kim

**Affiliations:** 1 Environmental Safety Group, Korea Institute of Science and Technology (KIST) Europe, 66123 Saarbrücken, Germany; cb.park@kist-europe.de (C.-B.P.); hpjeon@kist-europe.de (H.P.J.); 2 Lotte Fine Chemicals Co., Ltd., Ulsan 44714, Korea; mj0410@lottechem.com (M.J.S.); choi@lottechem.com (N.W.C.); sh0430.kim@lottechem.com (S.K.)

**Keywords:** aquatic-toxic effects, EU chemical regulation, glyoxal-containing cellulose ether

## Abstract

The objective of this study was to investigate the aquatic-toxic effects of glyoxal-containing cellulose ether with four different glyoxal concentrations (0%, 1.4%, 2.3%, and 6.3%) in response to global chemical regulations, e.g., European Union Classification, Labeling and Packaging (EU CLP). Toxicity tests of glyoxal-containing cellulose ether on 11 different microbial strains, *Microcystis aeruginosa*, *Daphnia magna*, and zebrafish embryos were designed as an initial stage of toxicity screening and performed in accordance with standardized toxicity test guidelines. Glyoxal-containing cellulose ether showed no significant toxic effects in the toxicity tests of the 11 freeze-dried microbial strains, *Daphnia magna*, and zebrafish embryos. Alternatively, 6.3% glyoxal-containing cellulose ether led to a more than 60% reduction in *Microcystis aeruginosa* growth after 7 days of exposure. Approximately 10% of the developmental abnormalities (e.g., bent spine) in zebrafish embryos were also observed in the group exposed to 6.3% glyoxal-containing cellulose ether after 6 days of exposure. These results show that 6.3% less glyoxal-containing cellulose ether has no acute toxic effects on aquatic organisms. However, 6.3% less glyoxal-containing cellulose ether may affect the health of aquatic organisms with long-term exposure. In order to better evaluate the eco-safety of cellulosic products containing glyoxal, further studies regarding the toxic effects of glyoxal-containing cellulose ether with long-term exposure are required. The results from this study allow us to evaluate the aquatic-toxic effects of glyoxal-containing cellulosic products, under EU chemical regulations, on the health of aquatic organisms.

## 1. Introduction

Cellulose ethers are water-soluble polymeric substances derived by the etherification of cellulose, which is one of the most widespread natural organic compounds. They are known as environmentally friendly polymers as a result of their properties (e.g., water solubility, pH stability, and biodegradation) [1,2,3,4,5]. Chemical modification of cellulose ethers has been performed to improve their physical and chemical properties (e.g., organic solubility, viscosity stability, water retention, and non-ionic charges), and cellulose ethers in mixed with organic or inorganic chemicals have also been produced to enhance the cellulosic product quality by physical blending of functionalized additives [2,5,6,7]. These derivatives can be used in a wide range of industrial applications, including construction products, ceramics, paints, personal care products, and pharmaceuticals [2,5,8,9]. With the increasing use of chemicals in industrial applications, the Globally Harmonized System of Classification and Labeling of Chemicals (GHS) proposed harmonized hazard communication elements, including safety data sheets (SDSs) for chemicals. It was adopted in the chemical regulations of the European Union (EU) Registration, Evaluation, Authorization, and Restriction of Chemicals regulation (REACH) and Classification, Labeling and Packaging (CLP) regulation [10,11]. Since most industrial applications of chemicals are based on mixtures containing more than a single chemical substance, the classification and labeling of chemical substances have been extended to a whole mixture of various substances from 2015 [11,12,13]. Therefore, SDSs for cellulosic products contained in functionalized additives under the EU CLP regulation are required.

Glyoxal is one of the most extensively used cross-linking agents in cellulosic products due to the advantage that its chemical reaction can enhance the solubility and dispersion of the polymer without chemical modification [6,7]. However, the glyoxal toxicity for human health is an issue and may cause sensitization for eye and skin contact, and its properties have led to the regulation of glyoxal content in functionalized products with glyoxal [6,14,15,16]. For example, the use of glyoxal in paper and textile wall coverings is prohibited [14]. The European Commission (EC) defined manufactured cosmetic products with a content of up to 100 mg/L glyoxal as safe [15,16]. When more than 1% of glyoxal is used for the reaction process with cellulose ether, companies are obliged to describe the cellulose ether products under EU CLP registration [6,15]. These regulations imply that the use and production of glyoxal-containing products may result in glyoxal being released into the environment and may thus arouse concern with respect to the potential impact on human and environmental health [15,16,17].

The predominant target compartments for glyoxal-containing cellulosic products in the environment are hydrospheres rather than sediment, when considering production to final emission [16,17]. Nevertheless, the aquatic hazards for glyoxal-containing cellulosic products are currently unclear, and the toxicity tests for these cellulosic products have been not performed for aquatic organisms. Cellulose is not classified as a hazardous polymer under the chemical regulations of the EU due to its being a natural organic compound [1,2,3,4,5,10,11]. Glyoxal is classified as a non-hazardous substance in the environment due to high half-effective concentrations of more than 100 mg/L in aquatic organisms, i.e., microorganisms, algae, *Daphnia*, and fish [16,17,18,19,20]. However, the eco-safety assessment of glyoxal-containing cellulosic products is urgently needed in response to the EU CLP regulations.

Therefore, the aim of this study was to provide information on aquatic toxicology available for glyoxal-containing cellulose ether based on a reliable evaluation of its hazardous properties. Surface-treated cellulose ethers with glyoxal were chosen in this study, since these glyoxal-containing cellulose ethers were specially developed to prevent lumping effects or to improve the rheological properties in wet blending applications, such as paints and emulsion. These cellulose ethers with four different concentrations of glyoxal were classified hazardous substances for human health by EU CLP regulations (Table 1). We evaluated the aquatic-toxic effects of glyoxal-containing cellulose ethers with four different concentrations by analysis of the toxicity tests, using four different aquatic organisms [21,22,23,24].

## 2. Materials and Methods

### 2.1. Test Samples

Glyoxal-containing cellulose ethers with four different glyoxal concentrations (0%, 1.4%, 2.3%, and 6.3%) were obtained from Lotte Fine Chemical Co., Ltd., Ulsan, Korea (cellulose, CAS 9004-65-3) (Table 2), and test solutions were prepared in accordance with the manufacturer’s manual (Lotte Fine Chemical Ltd., Ulsan, Korea) [25]. Briefly, surface-treated test samples in powder form were dissolved using a magnetic stirrer in purified cold water for 1 h at room temperature to ensure the complete removal of any undissolved powder samples before use in the toxicity tests with the different aquatic organisms.

#### 2.1.1. The Aquatic-Toxic Effects on Aquatic Organisms

**Figure 1:**
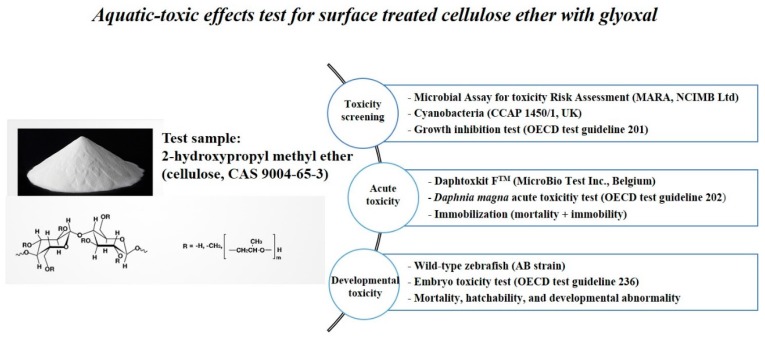
The sequential assessing of the aquatic-toxic effects of glyoxal-containing cellulose ethers on the various aquatic organisms according to the Organization for Economic Co-operation and Development (OECD) guidelines.

A schematic design for the aquatic-toxic effect tests is shown Figure 1. Releases to environment of the glyoxal-containing cellulose ethers are primary emissions to water due to high solubility [15,16,25]. The ecological effects on aquatic organisms may depend on ingestions or biodegradation of glyoxal-containing cellulose ethers [15,16]. However, the aquatic toxicity of glyoxal-containing cellulose ethers is unknown. To understand the aquatic toxicity on different biological levels in this study, the aquatic toxicity tests were conducted with four different aquatic organisms. 

Aquatic toxicity testing of glyoxal-containing cellulose ethers on 11 freeze-dried microbial strains, *Microcystis aeruginosa*, *Daphnia magna*, and zebrafish embryos have been covered by standardized methods [22,23,24,26]. The following points were investigated: the growth inhibition of microbial strains and cyanobacteria, *Daphnia* acute toxicity based on mortality and immobility, and zebrafish embryo toxicity based on mortality, hatchability, and abnormalities.

#### 2.1.2. Microbial Array for Toxicity Risk Assessment (MARA)

A MARA system involving 11 freeze-dried microbial strains was purchased from NCIMB Ltd., Aberdeen, UK (Table 3). The MARA for test solutions (i.e., glyoxal-containing cellulose ether with four different glyoxal concentrations) was performed according to the manufacturer instructions [26]. Briefly, 11 freeze-dried microbial strains were pre-incubated in a 96-well MARA plate with 150 µL of aqueous nutrient peptone (2% phytone peptone) for 4 h at 30 °C. After pre-incubation, 200 µL of the test solution was transferred to the 96-well MARA plate with 100 µL of medium containing 0.01% redox indicator. A volume of 15 µL of each microbial strain was added to each well and incubated for 18 h at 30 °C. After 18 h of incubation, the MARA plates were scanned by a scanner (HP Scanjet G4050, Hewlett Packard, Palo Alto, CA, USA) using transmitted light with a resolution of 100. The average growth rate of the 11 freeze-dried microbial strains exposed to test solutions was determined with the reduction of tetrazolium red using MARA software in quadruplicate.

#### 2.1.3. Algal Growth Inhibition Test

*Microcystis aeruginosa* was purchased from the Culture Collection of Algae and Protozoa, Cambria UK strain (CCAP 1450/1), and cultivated at 25 ± 1 °C with 16 h of light and 8 h of darkness (16L/8D) in Blue-Green medium (BG11, Sigma-Aldrich, Darmstadt, Germany) until use for the algal growth inhibition test (a total volume of ≥1 × 10^5^ cell/mL). Based on the finding in the MARA system that there was no influence between 11 freeze-dried microbial strains exposed to test solutions, except on 6.3% glyoxal-containing cellulose ethers, algal growth inhibition tests for 6.3% glyoxal concentrations only were conducted based on the protocol described in the OECD Guideline 201 [23,27].

#### 2.1.4. Daphnia Magna Acute Toxicity Test

*Daphnia magna* ephippia was obtained from Daphtoxkit F^TM^ (MicroBioTests Inc., Gent, Belgium) and was cultured according to the Daphtoxkit F^TM^ manual. Toxicity tests for four different glyoxal concentrations (0%, 1.4%, 2.3%, and 6.3%) of *Daphnia magna* neonates within 8 h after hatching were conducted in six-well cell culture plates (Cellstar^®^, greiner bio-one, Frickenhausen, Germany) filled with 10 mL of each test solution [28]. During the experimental period, *Daphnia magna* neonates were maintained at 23 ± 0.5 °C under a light cycle of 16 h light and 8 h darkness (16L/8D). The toxicity to *Daphnia magna* was investigated after an exposure period of 48 h to glyoxal-containing cellulose ether [22]. During the experimental period, the dead and immobilized individuals for each cellulose ether solution were recorded for calculating the immobilization (immobilization = (numbers of dead and immobilized individuals /number of initial individuals) × 100) after exposure to each cellulose ether solution. Tap water filtered through a Millipore^®^ 0.22 µm of nitrocellulose membrane (GSWP) filter (Merck KGaA, Darmstadt, Germany) after sterilization at 130 °C for 1 h was used as a control medium. Toxicity for each cellulose ether was determined in quadruplicate (10 daphnids per replicate).

#### 2.1.5. Zebrafish Embryo Toxicity Test

Wild-type zebrafish (AB strain) breeding and maintenance was performed under a long photoperiod (16L/8D light/dark cycle) at a 26.5 ± 0.5 °C water temperature [25,28]. Tap water filtered through a Millipore^®^ 0.22 µm GSWP filter (Merck KGaA, Darmstadt, Germany) after sterilization at 130 °C for 1 h was used as the control group. The zebrafish embryo toxicity tests with four different glyoxal concentrations (0%, 1.4%, 2.3%, and 6.3%) were conducted in six-well cell culture plates (Cellstar^®^, Greiner bio-one, Frickenhausen, Germany) filled with 10 mL of each test solution. Embryos at 8 h post-fertilization were treated with glyoxal-containing cellulose ether for 6 days which is commonly referred to as the embryonic stage. The mortality, hatchability, and developmental abnormalities in each test solution were recorded to assess the embryo toxicity of each glyoxal-containing cellulose ether [25,28]. The embryo toxicity for each cellulose ether was determined in quadruplicate (10 embryos per replicate).

#### 2.1.6. Statiscal Analysis

All errors are expressed as mean ± standard error of mean (SEM). Comparison between the toxicities for each glyoxal-containing cellulose ether was carried out using a post hoc Student–Newman–Keuls test in the one-way ANOVA (SigmaPlot version 12.5, Systat Software, Inc., San Jose, CA, USA). Statistical significance was set at *p* < 0.05.

#### 2.1.7. Ethical Statement

Tests for ecotoxicological effects of chemicals on algae, waterflea (*Daphnia magna*), and fish (*Danio rerio*) are covered by the OECD test guidelines for the testing of chemicals [22,23,24]. Toxicity tests with the zebrafish embryos were conducted in accordance with the protocol as prescribed by the OECD [24].

## 3. Results and Discussion

### 3.1. MARA and Cyanobacteria Algal Growth Inhibition

**Figure 2:**
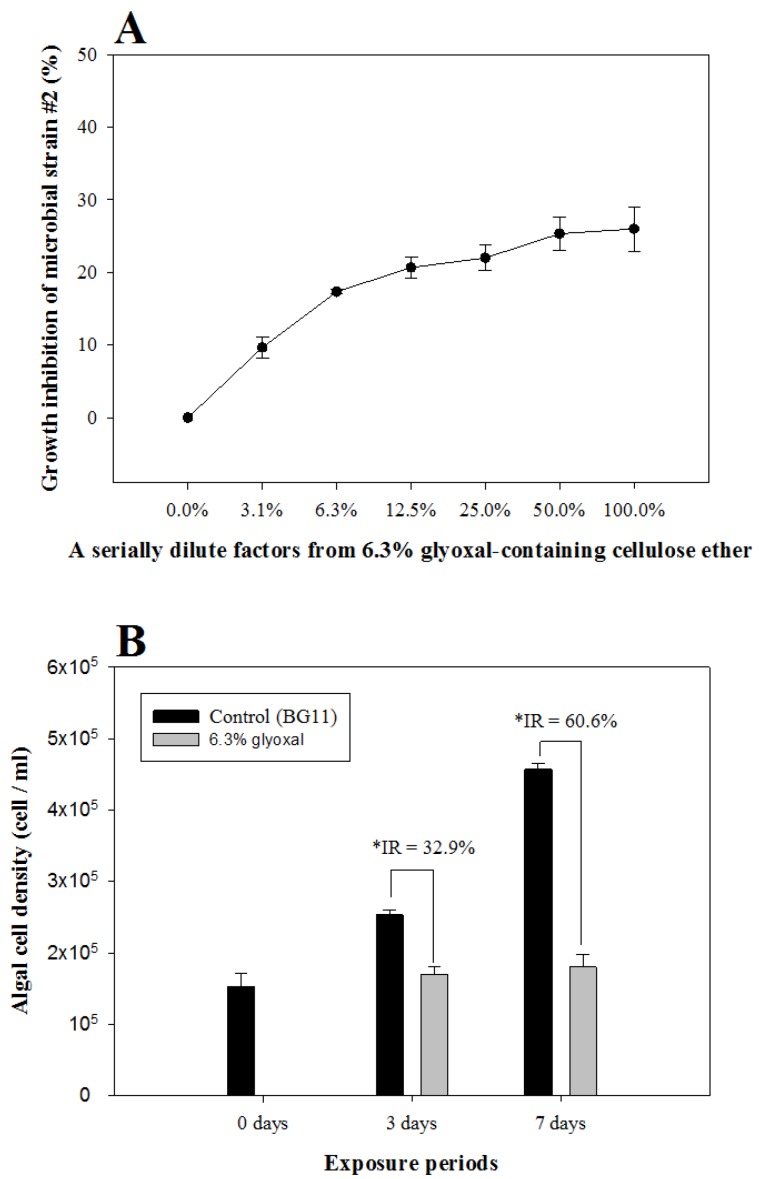
Growth inhibition rate of microbial strain #2 (**A**) and *M. aeruginosa* (**B**) after exposure to 6.3% glyoxal-containing cellulose ether. The inhibition rates (IR) of *M. aeruginosa* growth at 3 and 7 days after exposure to 6.3% glyoxal-containing cellulose ether were 32.9% and 60.6%, respectively. * denotes significant differences between algal cell density (*p* < 0.05).

The toxicity of each glyoxal-containing cellulose ether for 11 freeze-dried microbial strains and cyanobacteria are shown in Table 4 and Figure 2. There was no remarkable difference between the growth rates of the 11 freeze-dried microbial strains exposed to glyoxal-containing cellulosic products of ≤2.4%, due to the growth rates being more than 75% in all strains (Table 4). However, when exposed to glyoxal-containing cellulosic products of 6.3%, the growth rate of Microbial Strain #2 (*Brevundimonas diminuta*, NCIMB 30256) was less than 75% and tended to decrease in a dose-dependent manner (Table 4, Figure 2A). In addition, the growth inhibition rate of *M. aeruginosa* in the 6.3% glyoxal-containing cellulose ether-exposed group for 7 days was significantly higher (i.e., 60.6%) when compared to that of the group exposed for 3 days (a growth inhibition rate of 32.9%), and the decrease in growth was dependent on exposure time (Figure 2B). The difference between the microbial strains and *M. aeruginosa* in terms of the growth inhibition after exposure to glyoxal-containing cellulose ethers may be a result of exposure time rather than glyoxal concentration. These results imply that 6.3% glyoxal-containing cellulose ether may have bactericidal properties or may not be totally biodegradable over the short term, unlike in previous reports [1,2,3,4,5,15,16,17]. Therefore, the lack of effects on the aquatic microbial or bacterial growth may be due to the biodegradation of glyoxal-containing cellulose ether with exposure time. In order to examine the safety on aquatic microbial or bacterial growth, further study for examining the effect of biodegradation activity on aquatic microbial or bacterial growth is needed.

### 3.2. Daphnia Magna Acute Toxicity and Zebrafish Embryo Toxicity

**Figure 3:**
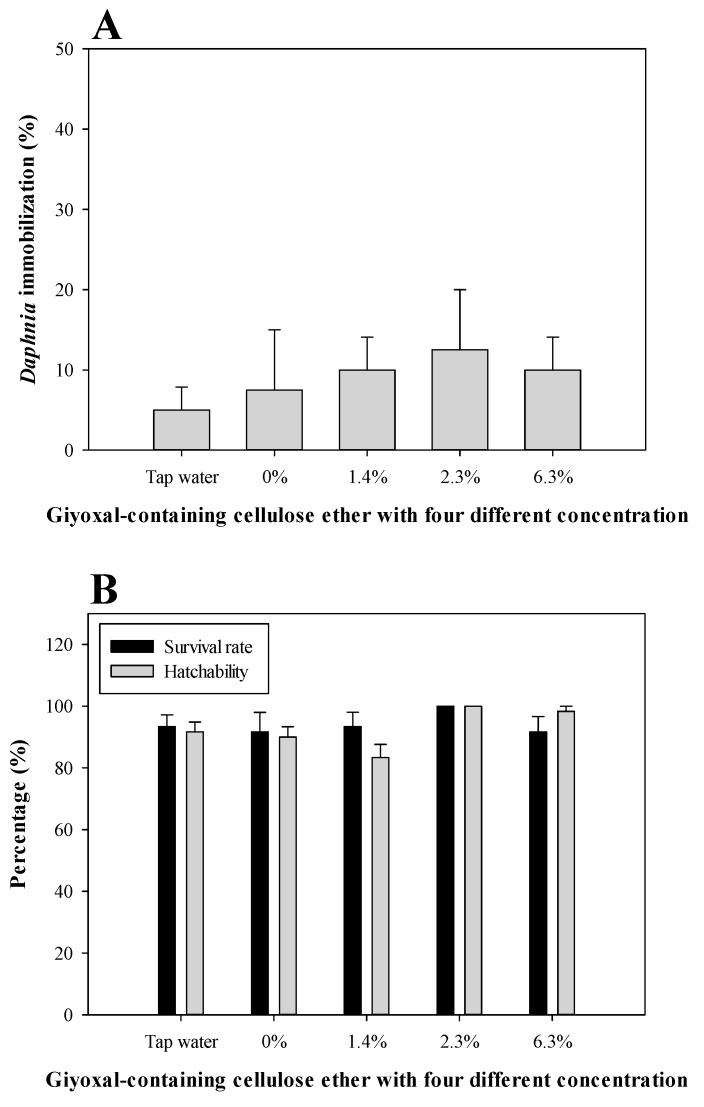
Acute toxicity of *Daphnia magna* after 48 h (**A**) and zebrafish embryo toxicity after 6 days (**B**) to glyoxal-containing cellulose ether with four different glyoxal concentrations after exposure.

After 48 h of exposure to the glyoxal-containing cellulose ethers with four different concentrations, the mortality and immobility of *Daphnia magna* were rarely observed in the glyoxal-containing cellulose ether-exposed group, due to the results that normal behavior was observed in more than 85% of cases in all groups (Figure 3A). The zebrafish embryo toxicity was also not observed in the glyoxal-containing cellulose ether-exposed group throughout the experimental period. The survival rate and hatching rate of zebrafish embryos exposed to glyoxal-containing cellulose ethers with four different concentrations were higher than 90% and 85%, respectively (Figure 3B). It appears that glyoxal-containing cellulose ethers had no effect on *Daphnia magna* acute toxicity or zebrafish embryo toxicity [16,17], even if 6.3% glyoxal-containing cellulose ether inhibited *M. aeruginosa* growth. The results indicate that the toxicity of glyoxal-containing cellulose ethers are sensitively reflected to *M. aeruginosa*, rather than waterflea (*Daphnia magna*) or zebrafish embryos [17,29].

**Figure 4:**
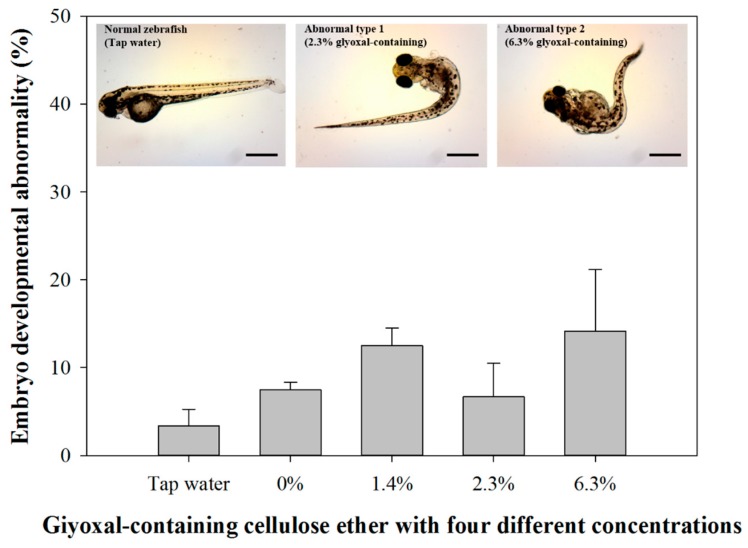
Zebrafish embryo toxicity to glyoxal-containing cellulose ether with four different glyoxal concentrations after 6 days of exposure. All scale bars are 500 µm.

Alternatively, while there was no acute toxicity of glyoxal-containing cellulose ether for the zebrafish embryos, developmental abnormalities, such as bent spines, were observed in the glyoxal-containing cellulose ether-exposed group, in particular 2.3% and 6.3% glyoxal-containing cellulose ethers (Figure 4). However, there were no significant differences between embryo developmental abnormalities among any of the groups (*p* > 0.05). It is possible that glyoxal-containing cellulose ethers do not result in acute toxicity in mortality and hatchability on zebrafish embryos, but may cause developmental abnormalities after 6 days of exposure. Unfortunately, studies examining the development toxicity of glyoxal-containing cellulose ethers on zebrafish embryos are rare and their toxicity mechanisms are also unknown. Further studies on the effects induced by glyoxal-containing concentration during zebrafish embryo development are therefore required.

Consequently, cellulose ethers with four different glyoxal concentrations have a lack of toxicity on different aquatic environments within the short term after exposure, suggesting that aquatic organisms are not taken in in the short term. However, in order to better understand the ecological safety of glyoxal-containing cellulose ethers, an evaluation of toxicity by long-term exposure on aquatic organisms is required.

## 4. Conclusions

In this study, toxicity tests using different aquatic organisms were conducted to investigate the effects of cellulosic products containing four different glyoxal concentrations (i.e., 0%, 1.4%, 2.3%, and 6.3%) as functional additives to aquatic environments under EU chemical regulations. A major finding from this study is the non-toxic effect of ≤6.3% glyoxal-containing cellulose ethers: even ≥1.4% glyoxal-containing cellulose ethers were classified as hazardous substances for human health by the EU CLP. Therefore, our systemic toxicity assessments might be valid for the risk assessment of glyoxal-containing cellulosic products under EU chemical regulations.

## Figures and Tables

**Table 1:** Hazardous classification of glyoxal-containing cellulose ethers with four different glyoxal concentrations calculated by the EU CLP.

Classification	Glyoxal-Containing Cellulose Ethers with Four Different Concentrations			
	0%	1.4%	2.3%	6.3%
Physical hazards	-	-	-	-
Health hazards	-	Skin sens. 1, Muta. 2	Skin sens. 1, Muta. 2	Skin sens. 1, Muta. 2
Environmental hazards	-	-	-	-

“-“ = Not calculable; “Skin sens. 1” = May cause an allergic skin reaction; “Muta. 2” = Suspected of causing genetic defects.

**Table 2:** Physicochemical characteristics of surface treated cellulose ethers with four different concentrations of glyoxal.

Test Sample	Viscosity (mPa·s)	pH	Moisture (%)	NaCl (%)	Methoxyl (%)	Hydropropoxyl (%)	Total-Glyoxal (%)
A	3.5	5.0–8.0	3	0.5	27–29	6–8	0
B							1.4
C							2.3
D							6.3

**Table 3:** Freeze-dried microbial strains for microbial assay for toxicity risk assessment (MARA, NCIMB Ltd., Aberdeen, UK).

MARA Number	Microbial Strain	
#1	*Microbacterium* sp.	(NCIMB 30255)
#2	*Brevundimonas diminuta*	(NCIMB 30256)
#3	*Citrobacter freundii*	(NCIMB 30257)
#4	*Comamonas testosteroni*	(NCIMB 30258)
#5	*Enterococcus casseliflavus*	(NCIMB 30259)
#6	*Delftia acidovorans*	(NCIMB 30260)
#7	*Kurthia gibsonii*	(NCIMB 30261)
#8	*Staphylococcus warneri*	(NCIMB 30262)
#9	*Pseudsomonas chlororaphis*	(NCIMB 30263)
#10	*Serratia rubra*	(NCIMB 30264)
#11	*Pichia anomola*	(NCIMB 30265)

**Table 4:** Average growth rate of freeze-dried microbial strains exposed to surface treated cellulose ethers with four different concentrations of glyoxal.

MARA Number	Average Values of Growth Rate ± SEM (*n* = 4)			
	Four Different Glyoxal Concentrations			
	0%	1.4%	2.3%	6.3%
#1	100.0 ± 0.0	100.0 ± 0.0	99.3 ± 0.8	100.0 ± 0.0
#2	86.3 ± 1.4	83.0 ± 0.8	77.5 ± 1.2	72.0 ± 3.1 **^†^**
#3	98.5 ± 1.2	96.5 ± 2.0	98.3 ± 0.9	92.3 ± 3.7
#4	100.0 ± 0.0	99.5 ± 0.5	99.8 ± 0.3	98.0 ± 1.2
#5	100.0 ± 0.0	100.0 ± 0.0	100.0 ± 0.0	79.3 ± 1.2
#6	98.8 ± 0.6	97.0 ± 0.8	87.8 ± 1.9	86.3 ± 1.9
#7	100.0 ± 0.0	100.0 ± 0.0	99.5 ± 0.5	100.0 ± 0.0
#8	98.5 ± 1.0	99.0 ± 0.6	91.0 ± 1.8	91.3 ± 2.0
#9	77.3 ± 1.9	77.5 ± 1.6	75.0 ± 2.0	86.7 ± 1.8
#10	95.8 ± 2.5	94.0 ± 2.2	93.3 ± 1.9	87.3 ± 1.8
#11	100.0 ± 0.0	100.0 ± 0.0	98.0 ± 2.0	98.7 ± 0.9

**^†^** Growth rate is lower than 75%, which was the lowest growth rate in this study.
